# Toward a Medication Information Literacy Indicator System for Older Adults: A Delphi Study

**DOI:** 10.1111/hex.14127

**Published:** 2024-06-28

**Authors:** Xiaoyu Zhou, Jia Yi, Li Bai, Mengyao Jiang, Wei Peng, Jing Liao, Hang Wang, Xiaorong Hou

**Affiliations:** ^1^ College of Medical Informatics Chongqing Medical University Chongqing China; ^2^ Department of Science and Education Hospital of Zigong Mental Health Central Sichuan China; ^3^ Department of Endocrinology The First Affiliated Hospital of Chongqing Medical University Chongqing China

**Keywords:** Delphi, indicator system, medication information literacy, older adults

## Abstract

**Background:**

The safety of medication use among older adults is a growing concern, given the aging population. Despite widespread attention, the exploration of medication literacy in older adults, particularly from the perspective of information literacy, is in its nascent stages.

**Methods:**

This study utilized the existing literature to define medication information literacy (MIL) as a theoretical framework. A two‐round Delphi survey was conducted to identify the essential components of a MIL indicator system for older adults. The analytic hierarchy process (AHP) was then used to assign weights to each indicator.

**Results:**

The study observed relatively high response rates in both rounds of the questionnaire, which, along with expert authority coefficients (Cr) of 0.86 and 0.89, underscores the credibility and expertise of the panellists. Additionally, Kendall's coefficient of concordance (Kendall's *W*) ranging from 0.157 to 0.33 (*p* < 0.05) indicates a consensus among experts on the identified indicators. Utilizing the Delphi process, a MIL indicator system for older adults was developed, comprising five primary and 23 secondary indicators. These indicators were weighted, with medication information cognition and acquisition emerging as pivotal factors in enhancing medication literacy among older adults.

**Conclusions:**

This study developed a MIL indicator system tailored for older adults using the Delphi approach. The findings can inform healthcare professionals in providing customized medication guidance and assist policymakers in crafting policies to enhance medication safety among older adults.

**Patient or Public Contribution:**

Patient and public engagement played a pivotal role in the development of our medication information literacy indicator system for older adults. Their involvement contributed to shaping research questions, facilitating study participation, and enriching evidence interpretation. Collaborations with experts in geriatric nursing, medicine, and public health, along with discussions with caregivers and individuals with lived experience, provided invaluable insights into medication management among older adults. Their input guided our research direction and ensured the relevance and comprehensiveness of our findings.

## 1 Introduction

China's aging population is rapidly increasing, presenting significant public health challenges [1]. In the recent national census of 2020, individuals aged 65 and above accounted for 13.5% of the total population, surpassing the UN's aging society benchmark of 7% [2]. This demographic shift places unprecedented pressure on the healthcare system, particularly concerning the safety of medication use among older adults [3, 4]. As they age, older adults experience a decline in physical function and an increase in chronic diseases, necessitating the management of multiple medications [5, 6, 7]. However, cognitive and sensory declines significantly hinder their ability to understand and remember essential medication information [8, 9]. This issue often leads to irrational medication behaviours, which can have severe consequences, including detrimental health outcomes, increased caregiver burden, and broader societal and policy challenges [10, 11, 12].

Further exploration reveals a significant contributor to such behaviours: a lack of medication literacy among patients [13, 14, 15]. Medication literacy encompasses individuals' abilities to obtain, comprehend, communicate, calculate, and process patient‐specific medication information, facilitating informed decisions about medication and health [13]. It is a specific aspect of health literacy that contains both levels of knowledge and ability and is an important predictor of correct medication‐taking behaviour in patients. While existing medication literacy assessments primarily focus on patients' mastery of medication expertise, there remains a noticeable gap in evaluating patients' medication information literacy (MIL)—their ability to effectively obtain, evaluate, and utilize medication information [16, 17]. This unaddressed aspect poses challenges within current assessment frameworks. Building upon the foundation laid by the Medical Library Association's definition of health information literacy [18], our study introduces the concept of MIL. It is a set of abilities for users to recognize medication information needs to familiarize themselves with possible information sources and apply them to retrieve relevant information; to evaluate the quality of medication information and its applicability in a specific situation; and to analyze, understand, and use medication information to make rational decisions about medication use.

Moreover, our examination of existing medication literacy assessment tools [19] revealed shortcomings in tools such as the Medication Literacy Questionnaire [20] and the Medication Literacy Assessment in Spanish and English scales [21], commonly used in the general population. These tools lack sufficient specificity for assessing medication literacy among older adults. Specifically, their measurement items do not adequately address the sensory and cognitive degeneration often experienced by this demographic, nor do they account for challenges in adapting to modern information platforms [22]. Hence, there is an urgent need for further research and development of MIL assessment tools tailored specifically to this age group.

In response to the identified obstacles, our study endeavours to address the medication literacy issues faced by older adults. Through the introduction of the concept of MIL and the development of tailored assessment tools, our aim is to bolster medication safety and enhance health outcomes within this demographic. This initiative will establish a standardized framework for systematically evaluating medication safety among older adults, thereby addressing a critical gap in current healthcare practices.

## 2 Methods

### 2.1 Study Design and Setting

**Figure 1:**
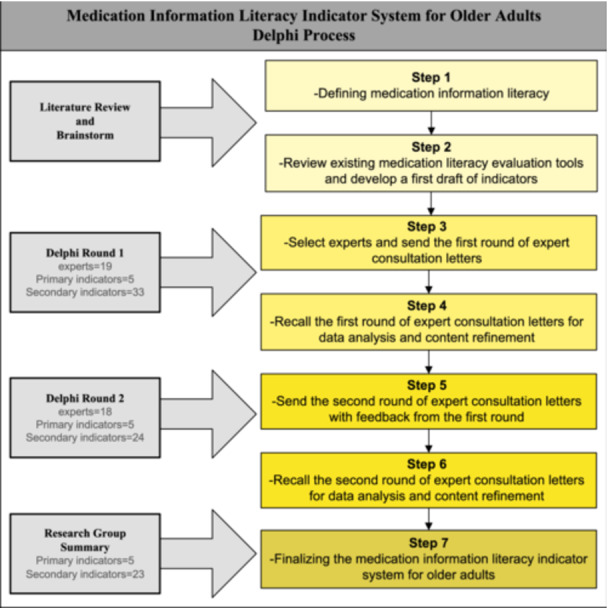
A two‐round Delphi process of constructing a medication information literacy indicator system for older adults.

MIL, which integrates the concepts of medication literacy and information literacy, was systematically explored to develop our study's framework. We conducted a comprehensive search using key terms and their synonyms, including ‘medication literacy’, ‘information literacy’, ‘medication use behaviours’, and ‘older adults’. We searched major databases, including PubMed, Web of Science, and China National Knowledge Infrastructure (CNKI), to gather studies from the inception of these databases up until March 2023. This extensive search resulted in the identification of approximately 282 studies that were pertinent to our research objectives. Several seminal studies have shaped our understanding of MIL among older adults. Wolf et al. elucidated the intricate relationship between health literacy and functional health status in older adults, shedding light on how literacy influences medication use behaviours [23]. Similarly, Davis et al. underscored the challenges posed by medication literacy issues among older adults, emphasizing the imperative for clearer medication information [24]. Additionally, Zhang et al. provided valuable insights into the development of tailored assessment tools for evaluating medication literacy in older adults, further enriching our comprehension of this domain [25]. Moreover, Mackert et al. investigated the interaction of older adults with health information technology, offering guidance on integrating information literacy within our framework [26]. Based on the insights gleaned from these studies and thorough discussions within our research group, a preliminary indicator system for MIL was established. Then, two rounds of expert consultation were conducted using the Delphi approach. On the basis of expert evaluation and suggestions, the indicator system was modified through internal discussion of the research group to form the final indicator system (Figure 1).

### 2.2 Panel Selection and Composition

In response to the interdisciplinary nature of our research, which spans geriatric nursing, information literacy, public health, and geriatric medicine, we meticulously selected experts with specialized knowledge and experience in these areas to ensure a comprehensive range of insights. Specifically, experts were chosen based on the following criteria: (1) Expertise in relevant fields: Panellists have proven expertise in geriatric nursing, information literacy, public health, or geriatric medicine, enabling them to provide informed perspectives on the multifaceted aspects of our research. (2) Experience and engagement: All experts have been actively engaged in their respective fields for at least 3 years, guaranteeing a depth of knowledge and experience that is essential for contributing effectively to our study. (3) Familiarity with the Delphi approach: Panellists possess a thorough understanding of the Delphi methodology, ensuring they can participate effectively in the consultation process and contribute constructively to refining our research framework. (4) Commitment to scientific rigour: The selected experts demonstrate a rigorous scientific approach and the ability to provide comprehensive and professional advice, enhancing the quality and validity of the consultation process.

In conclusion, the Delphi panel of experts comprised 19 members. Out of these 19 experts, nine were male, and 10 were female. Approximately 10 experts held professional titles above the deputy senior level, constituting 52.63% of the panel. Furthermore, 15 individuals possessed a master's degree or higher, accounting for 78.94% of the total. Among the experts, 10 (52.63%) were affiliated with educational and scientific research institutions, while nine (47.37%) were associated with medical and health institutions (refer to Table S1).

### 2.3 Generation of Expert Consultation Questionnaire

Having conducted a thorough review of pertinent literature, we proceeded to synthesize the dimensions of MIL. Subsequently, a preliminary indicator system was formulated through collaborative discussions within a research group. This indicator system comprised five primary indicators and 33 secondary indicators. Through deliberation, the first round of expert consultation questionnaires was formed. The questionnaire consisted of three parts: (1) Explanation of the questionnaire. The section included the research background, purpose and meaning; (2) Evaluation of each indicator by experts. In accordance with the Likert 5 scale, the importance and feasibility of the indicator were divided into five levels: ‘not important’ to ‘very important’ and ‘not feasible’ to ‘very feasible’, and they were assigned 1–5 points in turn. Experts were required to rate each indicator; they can make suggestions, such as addition and deletion of indicators, along with the reasons during this part; (3) Survey of expert' basic information to understand the experts' working years and fields. In this section, experts needed to assess their familiarity with the consulting content and the influence of different judgement bases (practical or scientific research experience, theoretical knowledge, domestic and foreign references, and visual judgement).

### 2.4 Implementation of Expert Consultation

From April to May 2023, we conducted two rounds of expert consultations involving 19 carefully selected experts in geriatric nursing, information literacy, public health, and geriatric medicine. The experts were chosen for their expertise, authority, and representativeness, ensuring their informed engagement and willingness to collaborate as consultants. The consultation process was executed through an email survey. Following the retrieval of the initial round of questionnaires, indicators failing to meet the following basic criteria were excluded: importance and feasibility assignment mean <3.5, coefficient of variation >0.25, and standard deviation >1 [27]. Subsequently, the indicator system underwent modification based on expert suggestions and discussions within the research group. The refined results were then presented to the experts for the second round of consultation. Ultimately, this iterative process led to the identification of five primary indicators and 23 secondary indicators.

### 2.5 Statistical Analysis

The collected data were processed in Microsoft Excel before being entered into a database. Subsequent data analysis utilized SPSS 26.0 (IBM Corporation, Armonk, NY, US). Descriptive statistics, including mean, standard deviation, and coefficient of variation, were employed to assess the importance and feasibility of each indicator.

The reliability of expert correspondence underwent meticulous evaluation utilizing objective criteria, encompassing three pivotal dimensions: authority, enthusiasm, and coordination [28]. Authority denotes the expertise and credibility demonstrated by experts within their respective domains. This was assessed through two primary factors: familiarity level (Cs), indicating the depth of the expert's knowledge and experience in the subject matter, and judgement basis (Ca), representing the criteria employed by the expert to evaluate the information. The authority coefficient (Cr) was computed as the arithmetic mean of Cs and Ca. An ideal Cr value exceeding 0.7 is considered indicative of robust authority [29]. Enthusiasm reflects the degree of active engagement and interest exhibited by experts in their involvement. It was quantified by analyzing the response rate of expert consultation forms, with a threshold of over 70% indicating substantial engagement the process [30]. Coordination signifies the level of consensus and harmony among experts in their evaluations and opinions. Quantitative assessment employed Kendall's coefficient, with statistical significance (*p* < 0.05) denoting reliability [31]. Higher coefficients indicate a stronger consensus among experts, thereby enhancing the reliability of the assessments.

To determine indicator weights, we employed the analytical hierarchy process (AHP), a decision‐making strategy that integrates both quantitative and qualitative assessment methods. This method involves systematically decomposing, quantifying, and synthesizing judgements to express subjective assessments in a numerical manner [32]. It also entails constructing a multilevel hierarchical structure model, establishing a judgement matrix, and conducting hierarchical ranking and consistency tests. In our study, we utilized Saaty's 1–9 scale to assign relative importance values to pairwise comparisons of each evaluation indicator. By comparing these relative importance values, we calculated the relative weights of each element within each level compared to the overall objective. Subsequently, we utilized the yaahp 12.8 software to compute weights using the AHP methodology, establishing a multilevel hierarchical structure model. We also conducted consistency tests, where a consistency index CR ≤ 0.1 indicates a reasonable construction of the hierarchical structure model [33]. It provides a reliable framework for determining indicator weights and supporting decision‐making processes.

## 3 Results

### 3.1 Literature Review to Compile a Preliminary Indicator System

A total of 57 relevant indicators were retrieved and extracted from the literature.

After careful consideration and discussion within the research group, a preliminary indicator system was established, comprising five primary indicators and 33 secondary indicators. This preliminary system formed the basis for subsequent Delphi consultations.

### 3.2 Round 1

The 19 invited participants agreed to participate and completed the first round of consultation, yielding a response rate of 100%. Among them, 13 experts provided suggestions, accounting for 68.42% of the total, indicating a high level of engagement among experts. According to the experts' self‐assessment, the familiarity coefficient (Cs) and judgement coefficient (Ca) were 0.79 and 0.94, respectively. Consequently, the degree of expert opinion authority (Cr) was calculated as 0.86 (Table 1). The degree of expert opinion coordination refers to whether the experts involved in the correspondence consultation disagree on the indicators, it is usually expressed by the coefficient of variation (CV) and Kendall's coefficient of concordance (Kendall's *W*). After the first round of expert consultation, the Kendall *W* values of indicators at all levels were 0.005–0.217 (Table 2).

**Table 1:** Degree of authority of experts.

	Round 1	Round 2
Degree of familiarity (Cs)	0.79	0.84
Coefficient of judgement (Ca)	0.94	0.94
Degree of expert opinion authority (Cr)	0.86	0.89

**Table 2:** Concordance coefficients of respondent experts.

	Round 1			Round 2		
	Kendall's *W*	*χ* ^2^	*p*	Kendall's *W*	*χ* ^2^	*p*
Primary indicators importance	0.005	0.369	0.985	0.157	11.27	0.024
Secondary indicators importance	0.157	95.44	<0.001	0.247	149.93	<0.001
Secondary indicators feasibility	0.217	89.77	<0.001	0.33	136.44	<0.001

Based on the definition of MIL, expert suggestions, and discussions within the research group regarding the five first‐level indicators and 33 second‐level indicators presented in the first round, a consensus was reached. This consensus involved retaining all first‐level indicators and 19 second‐level indicators for the second round, excluding 14 second‐level indicators that did not meet the basic conditions, and incorporating five additional second‐level indicators. Furthermore, we appropriately adjusted the content of indicators based on expert feedback.

For instance, the first‐level indicator ‘medication information demand’ was refined to ‘medication information cognition’, accompanied by a revised definition: ‘older adults recognizing the importance of medication information, expressing their demand for such information, and actively seeking medication‐related information’. Within the first‐level indicator ‘medication information acquisition’, the indicator ‘access to medication‐related information through multiple channels (such as popular science books, online media, medication instructions)’ was modified to ‘access to medication‐related information through mass communication channels (such as popular science books, television, medication instructions)’.

Ultimately, the revised indicator system, along with the data from all indicators in the first round, was forwarded to the second round for further consideration.

### 3.3 Round 2

A total of 18 experts completed the second round (response rate: 94.74%), of which 13 experts proposed amendments (72.2%). The experts' familiarity coefficient (Cs) was 0.840, and the judgement coefficient (Ca) was 0.940, that is, Cr was 0.890, which is higher than 0.70, indicating a high authority degree of experts (Table 1). After the second round of expert consultation, the Kendall *W* values were 0.157–0.33 (Table 2), which were statistically significant (*p* < 0.05), indicating that the overall level of expert coordination had been greatly improved.

In the second round of consultation, the importance and feasibility scores of the indicators met the necessary criteria. The number of first‐level indicators remained at five, while the second‐level indicators were streamlined to a total of 23. Notably, modifications were made to the indicator content. Specifically, under the first‐level indicator ‘medication information acquisition’, three indicators, such as ‘knowing the way to obtain medication information’, were consolidated into two indicators: ‘Be able to obtain medication‐related information through traditional media channels (e.g., popular science books, TV)’ and ‘Be able to obtain medication‐related information through new media channels (e.g., Tik Tok APP, WeChat official accounts)’.

### 3.4 Generation of MIL Indicator System for Older Adults

The Delphi process was conducted for research and evaluation of the five first‐level indicators and 33 second‐level indicators after constant revision and update of the indicators. The importance and feasibility scores of the indicators met the necessary criteria (see Table 3 for specific data). Ultimately, five first‐level indicators and 23 second‐level indicators were conclusively determined, constituting the MIL indicator system for older adults (refer to Table S2).

**Table 3:** Medication information literacy indicator system Delphi process results.

Primary indicators	Secondary indicators	Importance score (mean ± SD)	Importance coefficient of variation (CV)	Feasibility score (mean ± SD)	Feasibility coefficient of variation (CV)
Cognition	A1: recognize	4.78 ± 0.428	0.09	4.67 ± 0.594	0.13
	A2: know	4.94 ± 0.236	0.05	4.83 ± 0.514	0.11
	A3: express	4.67 ± 0.485	0.10	4.56 ± 0.511	0.11
	A4: have	4.58 ± 0.842	0.17	4.48 ± 0.845	0.18
	A5: willing	4.61 ± 0.698	0.15	4.61 ± 0.608	0.13
Acquisition	B1: obtain (traditional media)	4.83 ± 0.383	0.08	4.78 ± 0.428	0.09
	B2: obtain (new media)	4.83 ± 0.383	0.08	4.78 ± 0.548	0.11
	B3: seek	4.72 ± 0.575	0.12	4.61 ± 0.608	0.13
Understanding	C1: know	5.00 ± 0.000	0.00	4.78 ± 0.548	0.11
	C2: understand (dosage, route, interval)	4.89 ± 0.323	0.07	4.78 ± 0.548	0.11
	C3: understand (expiration date)	4.83 ± 0.383	0.08	4.67 ± 0.686	0.15
	C4: understand (health care professionals)	4.61 ± 0.502	0.11	4.33 ± 0.485	0.11
Evaluation	D1: assess	4.94 ± 0.236	0.05	4.44 ± 0.616	0.14
	D2: recognize	4.89 ± 0.323	0.07	4.28 ± 0.575	0.13
	D3: distinguish	4.89 ± 0.323	0.07	4.50 ± 0.618	0.14
	D4: select	4.37 ± 0.602	0.14	4.16 ± 0.774	0.18
Application	E1: summarize	4.17 ± 0.857	0.21	3.89 ± 0.676	0.17
	E2: take	4.94 ± 0.236	0.05	4.78 ± 0.428	0.09
	E3: observe	4.72 ± 0.461	0.10	4.50 ± 0.707	0.16
	E4: provide	4.61 ± 0.608	0.13	4.56 ± 0.705	0.15
	E5: store	4.94 ± 0.236	0.05	4.89 ± 0.323	0.07
	E6: clean	4.39 ± 0.698	0.16	4.56 ± 0.511	0.11
	E7: share	4.44 ± 0.616	0.14	4.33 ± 0.686	0.16

The AHP results revealed the following weight distribution for the five first‐level indicators, listed in descending order: medication information cognition (0.282), medication information acquisition (0.282), medication information understanding (0.163), medication information evaluation (0.163), and medication information application (0.110). The weights for each level index are outlined in Table 4. The consistency ratio (CR) for each judgement matrix was below 0.10, signifying strong consistency across all matrices.

**Table 4:** Medication information literacy indicator system weight calculation results.

Primary indicators	Weight	Secondary indicators	Weight	Combined weight
Cognition	0.2818	A1: recognize	0.2286	0.0644
		A2: know	0.3852	0.1085
		A3: express	0.1645	0.0464
		A4: have	0.0949	0.0268
		A5: willing	0.1267	0.0357
Acquisition	0.2818	B1: obtain (traditional media)	0.4000	0.1127
		B2: obtain (new media)	0.4000	0.1127
		B3: seek	0.2000	0.0564
Understanding	0.1631	C1: know	0.4118	0.0672
		C2: understand (dosage, route, interval)	0.2930	0.0478
		C3: understand (expiration date)	0.1872	0.0305
		C4: understand (health care professionals)	0.1080	0.0176
Evaluation	0.1631	D1: assess	0.4233	0.0691
		D2: recognize	0.2501	0.0408
		D3: distinguish	0.2501	0.0408
		D4: select	0.0764	0.0125
Application	0.1101	E1: summarize	0.0400	0.0044
		E2: take	0.2625	0.0289
		E3: observe	0.1726	0.0190
		E4: provide	0.1088	0.0120
		E5: store	0.2625	0.0289
		E6: clean	0.0584	0.0064
		E7: share	0.0951	0.0105

## 4 Discussion

### 4.1 Analysis of the MIL Indicator System for Older Adults

The indicator system utilized in this study is rooted in the concept of MIL as its theoretical framework. It comprises five primary indicators: medication information cognition, medication information acquisition, medication information understanding, medication information evaluation, and medication information application. Each of these indicators is tailored to the specific context of older adults, offering a comprehensive framework for assessing their MIL.

#### 4.1.1 Medication Information Cognition

The prevalence of cognitive impairment in older adults rises significantly with age [34]. Cognition, defined as the brain's ability to process, store, and extract information, plays a pivotal role in shaping health outcomes [35]. Previous studies have shown that the level of cognitive ability affects the health status of individuals to some extent [36, 37, 38]. For example, individuals with higher cognitive ability can be better aware of the importance of medication information, and their medication behaviour becomes scientific and safe. By contrast, individuals with lower cognitive ability are prone to miss and misuse medications, leading to an enormous risk of medication use safety for older adults. This study considers medication information cognition as a primary indicator, encompassing older adults' ability to recognize the importance of medication information, express their information needs, and actively seek relevant details. Under the cognitive dimension, our study considered the phenomenon of decreasing the expressive ability of older adults, and the A3 index particularly emphasized whether older adults can clearly express the daily medication information.

#### 4.1.2 Medication Information Acquisition

Studies on the information behaviour of older adults point out that older adults rely more on interpersonal communication in terms of information acquisition [39, 40]. However, their limited social range and homogeneity of social circles contribute to narrowed information access [41]. We included the examination of acquisition in the evaluation indicator, medication information acquisition refers to the ability of older adults to select appropriate sources of medication information, acquire medication information, and summarize the acquired medication information. In medication information acquisition. Recognizing the constraints on information channels for older adults [42], our indicator system scrutinizes medication information acquisition across traditional media, new media, and interpersonal interactions. The B1 indicator, rooted in traditional media channels (e.g., popular science books, TV), is exclusive to older age groups, while the B3 indicator reflects the social dynamics among older adults, who often seek information through interpersonal connections.

#### 4.1.3 Medication Information Understanding

In drug labels and drug instructions from medical personnel, the way medication information is arranged and presented often follows the logic of professional medical knowledge, and this seemingly meticulous logic differs from the cognitive logic of older adults' group. [43, 44] Moreover, key information, such as drug indications, adverse effects, and contraindications, are the guarantee of safe medication use in older adults. Thus, the ability to understand medication information should be tested in the indicator system. Medication information understanding means that older adults have a certain drug knowledge base and can understand the medication information they have obtained. This dimension also included secondary indicators of drug dose, intervals, and mode of administration.

#### 4.1.4 Medication Information Evaluation

Older adults have simpler social relationships, and their social networks shrink with age, they spend more time with people they trust and those who share common interests [45, 46]. Some studies showed that older adults judge the credibility of the information they receive on the basis of the closeness of their relationships with others, thus influencing the evaluation of the information [47, 48]. This underscores the need to scrutinize the ability of older adults to discern the reliability of medication information sources and evaluate the quality of medication‐related information. The primary metric for this evaluation is medication information evaluation, encompassing common aspects applicable across all age groups, such as the D1 indicator that assesses source reliability. Additionally, it addresses specific challenges prevalent in the older population, examining whether older adults can identify exaggerated descriptions in medication information (D2) and distinguish between medicines and health products (D3). These elements are integral to ensuring safe medication use in older adults.

#### 4.1.5 Medication Information Application

Older adults tend to have weak self‐management skills and unsafe medication use, such as missing medication or increasing medication dosage and frequency without authorization, thus affecting the therapeutic effect and may cause adverse drug reactions [49, 50]. Medication information application refers to the ability of older adults to apply the accurate medication information obtained to be able to solve the medication‐related problems they encounter. Older adults are at increased risk for a number of medication‐related problems such as adverse reactions and storing expired medications at home, due to taking multiple medications [51]. Therefore, we emphasized indicators E3 and E4, that is, whether older adults can observe their own adverse reactions and give feedback to healthcare professionals, and indicators E5 and E6, that is, whether older adults can properly store and regularly clean out expired medications.

### 4.2 Practical Application of the MIL Indicator System for Older Adults

The development of the MIL indicator system serves not only as a theoretical innovation but also as a practical tool aimed at assessing and enhancing the MIL of older adults. Through its application, healthcare providers gain valuable insights into and address the unique challenges that older adults face in managing their medications. Tailored educational initiatives, for example, can effectively improve cognition and comprehension of medication information within this demographic. Additionally, leveraging the system facilitates the creation of user‐friendly medication labels and instructions specifically designed to meet the cognitive and informational needs of older adults.

Moreover, the indicator system plays a pivotal role in shaping policy initiatives by emphasizing the need for accessible medication information channels and support structures tailored to the diverse needs of aging populations. Implementation of these measures can mitigate the incidence of medication errors, significantly enhancing the overall quality of healthcare for older adults.

In conclusion, our primary objective is to assess and enhance the MIL of older adults, ultimately improving their medication safety. By identifying barriers to effective medication information management within this demographic and proposing evidence‐based solutions, our study contributes to a nuanced understanding of the challenges and opportunities in enhancing medication safety for this group. Through practical application, MIL indicator system holds promise for improving healthcare outcomes and ensuring the well‐being of older adults.

### 4.3 Strengths and Limitations

The strength of this study is that the physical, psychological, and social relationship characteristics of older adults were integrated into the formulation of the indicators. Although the experts involved in this study have certain academic representativeness in their fields, the Delphi approach, as a qualitative method, cannot completely exclude the influence of experts' subjective factors, and biases may exist in the selection of some indicators. Consequently, to advance this research, we intend to utilize this indicator system as the foundation for the subsequent phase. This involves preparing a questionnaire based on the understanding habits of older adults, conducting an investigation into the MIL of the older population, and undertaking an empirical study with reliability analysis to further validate the rationale behind the indicators.

## 5 Conclusions

Medication safety for older adults will continue to be a concern in the foreseeable future. MIL plays a critical role in ensuring the safe use of medication among older adults. Despite this, research on MIL remains scarce. This study addresses this gap by constructing an indicator system for evaluating the MIL of older adults using the Delphi approach. The system identifies specific weights for each indicator, enabling a more comprehensive evaluation of the factors influencing MIL levels. Healthcare professionals can use this system to provide personalized medication guidance to older adults based on their individual levels of medication literacy. Additionally, policymakers can use this system to gain a deeper understanding of the current medication use situation among older adults and formulate medication safety promotion policies from a new perspective.

In navigating the evolving healthcare landscape characterized by an aging demographic and advancing technology, enhancing MIL in older adults becomes increasingly crucial. This study not only bridges existing research gaps but also sets the stage for future initiatives to foster a safer and more informed medication environment for older adults.

## Author Contributions

**Xiaoyu Zhou:** investigation, writing–original draft, visualization, validation, conceptualization, writing–review and editing. **Jia Yi:** conceptualization, investigation, writing–original draft. **Li Bai:** conceptualization, investigation, writing–review and editing. **Mengyao Jiang:** investigation. **Wei Peng:** investigation, conceptualization. **Jing Liao:** investigation. **Hang Wang:** investigation. **Xiaorong Hou:** conceptualization, investigation, funding acquisition, writing–original draft, writing–review and editing.

## Ethics Statement

The research protocol was approved by the Ethics Committee of Chongqing Medical University under reference number 2018011.

## Conflicts of Interest

The authors declare no conflicts of interest.

## Supporting information

Supporting information.

Supporting information.

## Data Availability

The data that supports the findings of this study are available in the Supporting Information material of this article.
