# Supplementary material for: Toward a Medication Information Literacy Indicator System for Older Adults: A Delphi Study
Source: Health Expect. 2024 Jun 28;27(4):e14127. doi: 10.1111/hex.14127 (PMC11212333; doi:10.1111/hex.14127)
Supplement: Supplementary file 1 — Supporting information. [file HEX-27-e14127-s002.doc]

**Supplement Table S1.** Demographic overview of experts

| Demographics | Count (%) |
| --- | --- |
| Gender, n (%) |  |
| Female | 10 (53) |
| Male | 9 (47) |
| Professional Experience, n (%) |  |
| ≤10 years | 7 (37) |
| 10-20 years | 7 (37) |
| 20-30 years | 4 (21) |
| ≥30 years | 1 (5) |
| Degree, n (%) |  |
| PhD | 5 (26) |
| Master's | 10 (53) |
| Bachelor's | 4 (21) |
| Professional Title, n (%) |  |
| Full Professor | 4 (21) |
| Associate Professor | 6 (32) |
| Senior Lecturer | 5 (26) |
| Junior Lecturer | 4 (21) |
| Research Fields, n (%) |  |
| Geriatric Nursing | 4 (21) |
| Geriatric Medicine | 4 (21) |
| Public Health | 6 (32) |
| Information Literacy | 5 (26) |
