# Supplementary material for: Toward a Medication Information Literacy Indicator System for Older Adults: A Delphi Study
Source: Health Expect. 2024 Jun 28;27(4):e14127. doi: 10.1111/hex.14127 (PMC11212333; doi:10.1111/hex.14127)
Supplement: Supplementary file 2 — Supporting information. [file HEX-27-e14127-s001.doc]

**Supplement Table S2.** The system: Medication information literacy indicator system for older adults.

| **Primary indicators** | **Secondary indicators** |
| --- | --- |
| Medication Information  Cognition | A1 Recognize the importance of medication information for medication use (e.g., indications, adverse effects, contraindications) |
|  | A2 Know their own medication information needs in daily medication use (e.g., route of administration, dose, interval between medications) |
|  | A3 Be able to clearly express the needs of their own health condition for medication information in the daily use of medication |
|  | A4 Have the awareness of actively seeking medication information |
|  | A5 Willing to accept medication guidance from professionals (pharmacists, health care professionals) in daily life |
| Medication Information Acquisition | B1 Be able to obtain medication-related information through traditional media channels (e.g., popular science books, TV) |
|  | B2 Be able to obtain medication-related information through new media channels (e.g., Tik Tok APP, WeChat official accounts) |
|  | B3 Be able to actively seek help from others to find the medication information needed |
| Medication Information Understanding | C1 Know the indications, adverse effects, and contraindications of the medication taken through guidance of medical personnel or by reading the instruction manual |
|  | C2 Understand the dosage, route of administration, and interval of the medication taken by reading the drug label or drug instructions |
|  | C3 Be able to understand the expiration date of the medication by reading the drug label or the drug package |
|  | C4 Understand the answers provided by healthcare professionals regarding medication use |
| Medication Information  Evaluation | D1 Be able to assess the reliability of sources providing medication information |
|  | D2 Recognize inaccurate or exaggerated descriptions in medication information |
|  | D3 Be able to distinguish between medicines and health products |
|  | D4 Be able to select the medication information they need from a large amount of information |
| Medication Information Application | E1 Be able to summarize and organize the acquired knowledge of medication information in a timely and correct manner |
|  | E2 Be able to take medication correctly according to medication guidance information |
|  | E3 Be able to observe their own reactions after taking medication and compare them with the adverse reactions in the drug instructions |
|  | E4 Be able to provide feedback to healthcare professionals on medication guidance (e.g., adverse reactions after taking medication) when seeing a doctor again |
|  | E5 Be able to store medications properly according to medication information, such as protection from light and moisture |
|  | E6 Be able to stock up on medications according to their information and regularly clean up expired medications |
|  | E7 Be able to share medication information with others in a timely manner |
